# Obesity reduced survival with 5-fluorouracil and did not protect against chemotherapy-induced cachexia or immune cell cytotoxicity in mice

**DOI:** 10.1080/15384047.2022.2108306

**Published:** 2022-08-13

**Authors:** Brandon N. VanderVeen, Thomas D. Cardaci, Sierra J. McDonald, Sarah S. Madero, Christian A. Unger, Brooke M. Bullard, Reilly T. Enos, Kandy T. Velázquez, Jason L. Kubinak, Daping Fan, E. Angela Murphy

**Affiliations:** a Department of Pathology, Microbiology, and Immunology, University of South Carolina School of Medicine – Columbia, Columbia, SC, USA; b Department of Cell Biology and Anatomy, University of South Carolina School of Medicine – Columbia, Columbia, SC, USA

**Keywords:** Chemotherapy, inflammation, skeletal muscle, adipose tissue, liver toxicity

## Abstract

Fluorouracil/5-flourouracil (5FU) is a first-line chemotherapy drug for many cancer types; however, its associated toxicities contribute to poor quality of life and reduced dose intensities negatively impacting patient prognosis. While obesity remains a critical risk factor for most cancers, our understanding regarding how obesity may impact chemotherapy’s toxicities is extremely limited. C56BL/6 mice were given high fat (Obese) or standard diets (Lean) for 4 months and then subjected to three cycles of 5FU (5d-40 mg/kg Lean Mass, 9d rest) or PBS vehicle control. Shockingly, only 60% of Obese survived 3 cycles compared to 100% of Lean, and Obese lost significantly more body weight. Dihydropyrimidine dehydrogenase (DPD), the enzyme responsible for 5FU catabolism, was reduced in obese livers. Total white blood cells, neutrophils, and lymphocytes were reduced in Obese 5FU compared to Lean 5FU and PBS controls. While adipocyte size was not affected by 5FU in Obese, skeletal muscle mass and myofibrillar cross section area were decreased following 5FU in Lean and Obese. Although adipose tissue inflammatory gene expression was not impacted by 5FU, distinct perturbations to skeletal muscle inflammatory gene expression and immune cell populations (CD45^+^ Immune cells, CD45^+^CD11b^+^CD68^+^ macrophages and CD45^+^CD11b^+^Ly6c^lo/int^ macrophage/monocytes) were observed in Obese only. Our evidence suggests that obesity induced liver pathologies and reduced DPD exacerbated 5FU toxicities. While obesity has been suggested to protect against cancer/chemotherapy-induced cachexia and other toxicities, our results demonstrate that obese mice are not protected, but rather show evidence of increased susceptibility to 5FU-induced cytotoxicity even when dosed for relative lean mass.

## Introduction

The prevalence and incidence of cancer and the number of patients receiving chemotherapy have increased over the past several decades.^1^ While novel and efficacious cancer therapies continue to emerge, traditional cytotoxic chemotherapies remain at the forefront of anticancer strategies.^2^ Unfortunately, chemotherapeutics have pervasive off-target effects concomitant with patients developing chemoresistance which hamper efficacy.^3^ Among these side effects are reduced blood counts, debilitating fatigue and weakness, and cachexia – the loss of lean mass secondary to disease.^4–6^ In addition to the underlying cancer, patients often have preexisting conditions and co-morbidities that hold the potential to exacerbate these off-target effects.^7,8^ Unfortunately, inherent difficulty in studying these converging conditions has led to a dearth of investigations examining the implications of preexisting comorbidities on cytotoxic chemotherapies.

5 fluorouracil (5FU), either alone or in combination treatment, is a first-line therapy for colon, breast, head and neck, and pancreatic cancers.^2^ Improved understanding of 5FU has led to superior strategies to enhance its therapeutic efficacy; however, much less is known about its off-target effects.^9–11^ 5FU is an antimetabolite, a uracil analogue, that incorporates into DNA/RNA to inhibit proliferation through inhibiting thymidylate synthase activity.^12,13^ Given its direct impact on proliferating cells, 5FU inhibits cancer cell growth, but with little specificity. While 5FU is cytotoxic, when catabolized in the liver by the dihydropyrimidine dehydrogenase (DPD) enzyme, it is excreted in the urine as nontoxic α-fluoro-β-alanine.^13^ However, patients with DPD deficiency have been demonstrated to experience severe toxicities with a prolonged 5FU half-life.^14^

Currently, over 40% of American adults are obese and obesity is linked to an increased risk for 13 different cancer types including breast and colorectal cancers.^15–17^ Given this, obese individuals are increasingly more likely to undergo chemotherapy. Obesity negatively impacts metabolic plasticity and homeostasis as well as overall immune health.^18,19^ Consequently, between 50% and 90% of obese patients have nonalcoholic fatty liver disease (NAFLD), which disrupts liver enzymes resulting in impaired or altered drug metabolism.^20,21^ However, the impact of NAFLD on chemotherapeutic drug metabolism and consequential toxicity has been vastly understudied. Yet, it has been speculated that the increased cancer mortality that is associated with an obese state is in part attributable to the undertreatment of the patient.^22^ While our understanding of 5FU’s off-target effects is improving, significant gaps remain contributing to the continued reduction in quality of life and subsequent dose modifications, especially in the context of an obese state. The purpose of the current study was to examine the impact of obesity on the toxicities of chemotherapy in liver, adipose, and skeletal muscle tissues as well as circulating immune cells.^23^ While several dosing strategies have been suggested (e.g. body weight, body surface area, and lean mass), we aimed to dose all mice based on lean mass to minimize risk of overdosing obese mice (~90% greater body weight, ~45% greater BSA, and ~30% greater lean mass). We hypothesized that while an obesity paradox has been postulated,^24,25^ the susceptibility to 5FU-induced toxicities would be greater in obese mice even when dosed for lean mass.

## Results

### Obesity exacerbated body weight loss and decreased survival with 5FU

**Figure 1.**
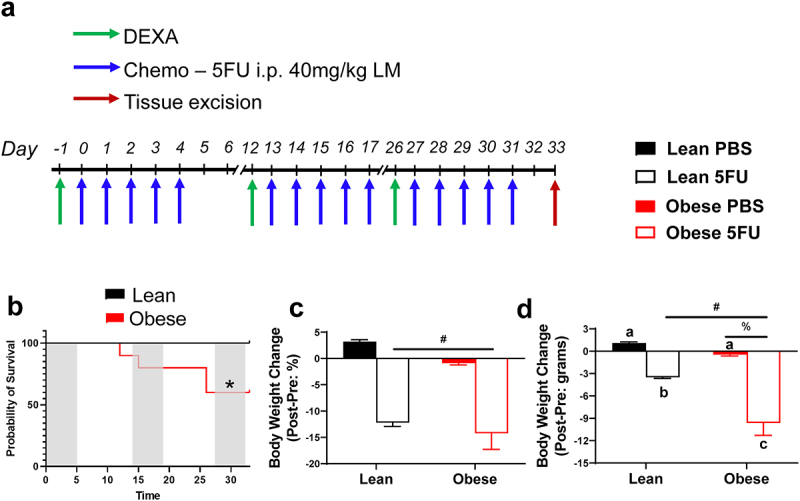
**Experimental design, survival, and body weight change with 5 fluorouracil treatment**. a) Experimental timeline. Mice underwent dual-energy x-ray absorptiometry (DEXA) analysis prior to each 5FU dosing cycle on days −1, 12, and 26. Mice were given 5 i.p. injections of either 40 mg/kg lean mass of 5FU dissolved in PBS or empty PBS control. Mice were euthanized 48 hours following the last dose of 5FU/PBS. b) Probability of survival in lean and obese mice given 3 cycles of 5FU. c) Absolute body weight change given in grams. Values are given as *Pre* – *Post*. d) Relative body weight change given in percent from baseline. Values are given as (*Pre* – *Post*)/*Pre*. Values are means ± SEM. Two-way ANOVA and LSD post hoc and multiple comparisons. *Indicates significant difference in survival. #Indicates main effect of 5FU. %Indicates main effect of Obese. Different letters signify statistically significant differences with an interaction. Significance was set as *p* < .05.

As expected, there were main effects (p < .0001) of Obese to have increased body weight by 89.5%, body surface area by 46.3%, and lean mass by 33.5% prior to the initiation of treatment (Table 1). Mice were then randomized and subjected to three cycles of 5FU or PBS (Figure 1a). Survival probability was assessed, and unexpectedly, obesity significantly (p = .025) reduced survival; indeed, 40% (n = 4/10) of Obese mice could not complete 3 cycles of 5FU and were euthanized for tissue collection once they reached >15% BW loss per University of South Carolina IACUC guidelines. All Lean 5FU animals (100%; n = 5/5) – and PBS controls (n = 15/15) – survived 3 cycles consistent with our previous findings (Figure 1b).^10^ As we expected, there were main effects of 5FU to induce relative body weight loss (p < .0001; %); (Figure 1c) and total body weight loss (p < .0001; grams;) (Figure 1d). Interestingly, there was a main effect of Obese to have increased total body weight loss (p = .002; grams) (Figure 1d); a significant interaction (p = .05) was detected and revealed that within 5FU, Obese had significantly greater body weight loss compared to Lean (Figure 1d).

**Table 1.** Animal characteristics prior to each 5FU dosing cycle.

		Lean	Obese	Lean v Obese Delta (%)	*Time [X]* v Pre 1 Delta (%)	
Outcome	Time				Lean	Obese
BMD (g/cm^2^)	*Pre 1*	0.0550 ± 0.0010	0.0493 ± 0.0005	−10.3	-	-
	*Pre 2*	0.0584 ± 0.0008	0.0529 ± 0.0006	−9.4	6.2	7.3
	*Pre 3*	0.0586 ± 0.0010	0.0509 ± 0.0005	−13.2	6.5	3.1
BMC (g)	*Pre 1*	0.440 ± 0.010	0.452 ± 0.007	2.7	-	-
	*Pre 2*	0.518 ± 0.011	0.485 ± 0.011	−6.3	17.7	7.4
	*Pre 3*	0.538 ± 0.013	0.465 ± 0.010	−13.5	22.3	3.0
Bone Area (cm^2^)	*Pre 1*	7.97 ± 0.10	9.15 ± 0.11	14.7	-	-
	*Pre 2*	8.87 ± 0.16	9.17 ± 0.23	3.5	11.2	0.3
	*Pre 3*	9.18 ± 0.08	9.16 ± 0.23	−0.2	15.1	0.1
Lean (g)	*Pre 1*	23.3 ± 0.8	31.1 ± 0.5	33.5	-	-
	*Pre 2*	23.7 ± 0.7	29.2 ± 0.4	23.5	1.5	−6.2
	*Pre 3*	21.9 ± 0.9	29.2 ± 0.8	33.2	−6.0	−6.2
Fat (g)	*Pre 1*	4.3 ± 0.5	22.6 ± 0.5	421.7	-	-
	*Pre 2*	4.1 ± 0.3	19.5 ± 0.4	379.7	−6.5	−14.0
	*Pre 3*	3.2 ± 0.5	19.5 ± 1.0	514.3	−26.7	−13.7
BSA (cm^2^)	*Pre 1*	97.2 ± 0.8	142.2 ± 1.1	46.3	-	-
	*Pre 2*	90.8 ± 1.0	135.1 ± 2.5	48.8	−6.6	−5.0
	*Pre 3*	87.5 ± 1.2	135.4 ± 2.2	54.7	−10.0	−4.8
Body Weight (g)	*Pre 1*	30.4 ± 0.5	57.6 ± 1.2	89.5	-	-
	*Pre 2*	28.1 ± 0.7	51.0 ± 2.1	81.5	−7.6	−11.5
	*Pre 3*	26.6 ± 0.9	51.2 ± 1.9	92.5	−12.5	−11.1

Notes: Values are means ± SEM. Bone mineral density given in grams/centimeters,^2^ bone mineral content given in grams (g), bone area given in centimeters,^2^ lean mass given in grams (g) and fat mass given in grams (g) analyzed via dual-energy x-ray absorptiometry (DEXA) prior to each 5FU dosing cycle. Lean n = 5 for each time point. Obese n = 10 for “Pre 1”, n = 8 for “Pre 2”, n = 6 for “pre 3”. Body surface area calculated using Meeh’s equation (BSA = *k* mass^0.667^) where *k* = 9.822.

### Obesity increased susceptibility to 5FU-induced cytopenia

**Figure 2.**
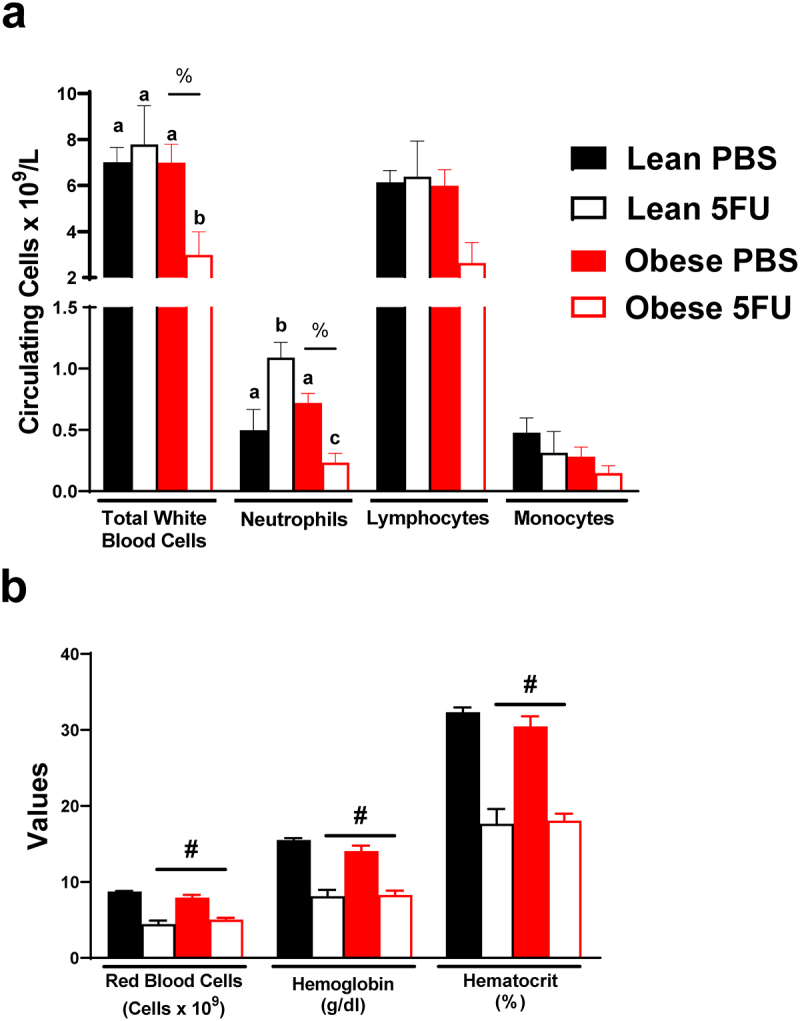
**Blood counts**. a) Circulating total white blood cells and white blood cell subsets – neutrophils, lymphocytes, and monocytes given as the number of cells x10^9^**/**L. b) Circulating red blood cells given as number of cells x10^12^**/**L. Circulating hemoglobin counts given as grams/dL. Hematocrit percentage (%). Values are means ± SEM. Two-way ANOVA and LSD post hoc and multiple comparisons. #Indicates main effect of 5FU. %Indicates main effect of Obese. Different letters signify statistically significant differences with an interaction. Significance was set as *p* < .05.

Next, we sought to examine the impact of obesity on the established deleterious effects of 5FU on blood cell counts – a key clinical outcome for dose intensity modifications. There was a main effect (p = .036) of Obese to have reduced white blood cells. Additionally, a significant interaction (p = .038) revealed that within Obese, 5FU had reduced white blood cells compared to PBS (Figure 2a) and that within 5FU, Obese had decreased white blood cells compared to Lean. Similarly, there was a main effect (p = .007) of Obese to have reduced circulating neutrophils (Figure 2a). A significant interaction (p < .0001) revealed that within Obese, 5FU had reduced neutrophils compared to PBS (Figure 2a), and within 5FU, Obese neutrophils were decreased compared to Lean (Figure 2a). Interestingly, it was also revealed that within Lean, 5FU increased neutrophils compared to PBS; however, one cycle of 5FU induced neutropenia as previously demonstrated (Supplemental Figure 1).^9^ Total lymphocytes followed a similar trend as total white blood cells and neutrophils; however, this did not achieve statistical significance (main effect of Obese – p = .055; interaction – p = .07; Figure 2a). Circulating monocytes did not show any semblance of statistical differences (Figure 2a). It was also observed that one cycle of 5FU decreased total white blood cells, neutrophils, and lymphocytes, regardless of weight status (Supplementary figure 1). Last and as expected, there were main effects (p < .0001) for 5FU to decrease red blood cells, hemoglobin, and hematocrit regardless of weight status (Figure 2b). Together, these results highlight that an obese phenotype prolongs susceptibility to 5FU-induced cytopenia and did not protect against 5FU-induced anemia.

### Obesity induced NAFLD and liver dihydropyrimidine dehydrogenase expression

**Figure 3.**
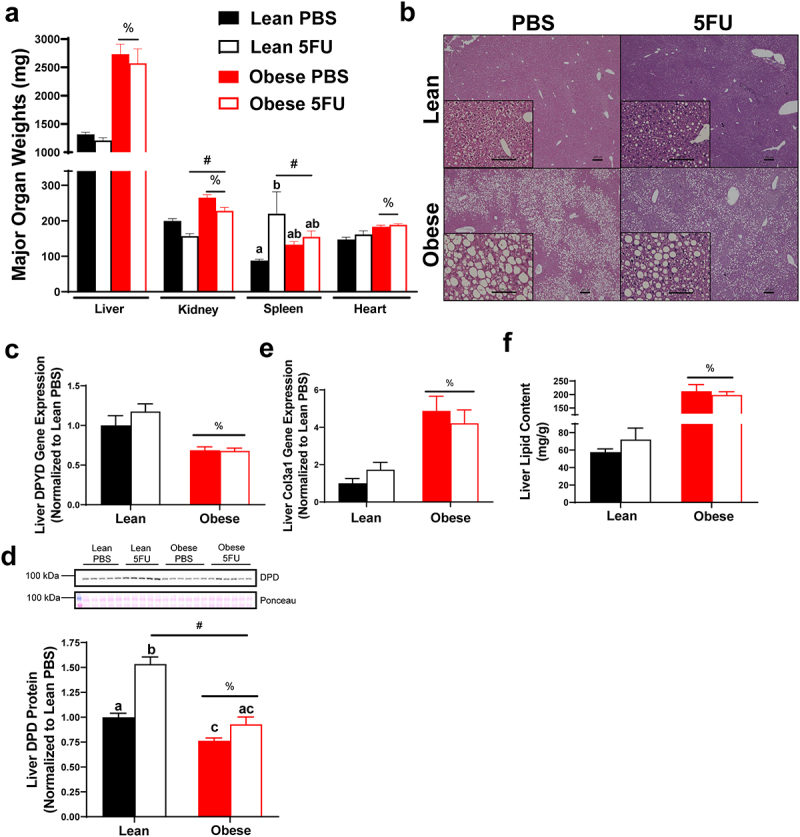
**High fat diet-induced liver dysfunction associated with obesity**. a) Organ weights excised from mice and euthanasia. b) Liver sections were stained with Hematoxylin and Eosin. Representative images are 4x with 20x inserts demonstrating signs of nonalcoholic fatty liver disease. Scale bars are 100 μm (20x) and 200 μm (4x). c) Liver gene expression of dihydropyrimidine dehydrogenase (DPYD) and d) collagen type 3 alpha 1 (Col3a1). Gene values were normalized to vehicle treated controls and compared to five reference targets. e) Liver lipid content measured using Folch’s extraction method in frozen liver. f) Quantified liver protein expression of DPD and western blot images. Values are means ± SEM. Two-way ANOVA and LSD post hoc and multiple comparisons. #Indicates main effect of 5FU. %Indicates main effect of Obese. Different letters signify statistically significant differences with an interaction. Significance was set as *p* < .05.

Based on the reduced survival and exacerbated cytopenia, we sought to examine the impact of obesity on liver health given its role in 5FU metabolism. There was a main effect (p < .0001) of Obese to have increased liver weight (Figure 3a), concomitant with signs of NAFLD (Figure 3b). We also observed main effects of Obese to have increased kidney (p < .0001) and heart (p < .0001) weights and a main effect (p = .0003) of 5FU to have decreased kidney weight (Figure 3a). Interestingly, we observed a main effect (p = .0057) of 5FU to have increased spleen weight; this was largely due to the increase seen in the Lean 5FU group as an interaction (p = .04) showed that within Lean, 5FU had increased spleen weight compared to PBS (Figure 3a) but this same effect was not observed for Obese 5FU.

There was a main effect (p < .0001) of Obese to have reduced liver gene expression of dihydropyrimidine dehydrogenase (*dpyd*) irrespective of 5FU treatment (Figure 3c). To corroborate the *dpyd* gene expression data, we found a main effect (p < .0001) of Obese to have reduced DPD protein expression and a main effect (p < .0001) of 5FU to increase DPD. A significant interaction (p = .004) revealed that within PBS, Obese reduced DPD protein compared to Lean, and within 5FU, Obese was reduced compared to Lean, whereas within Lean, 5FU increased DPD protein compared to PBS (Figure 3d).

Additionally, there was a main effect (p = .0003) of Obese to have increased gene expression of liver collagen, *col3a1* (Figure 3e), and a main effect (p < .0001) of Obese to have increased total liver lipid content (Figure 3f) irrespective of 5FU treatment, further demonstrating high fat diet-induced NAFLD. Our obese mice showed signs of metabolic dysfunction with a main effect (p < .0001) of Obese to have elevated circulating insulin, whereas chemotherapy treated mice showed reduced insulin evidenced by a main effect (p = .008) of 5FU (Supplemental figure 2).

### 5FU decreases muscle mass and myofibrillar cross sectional area in both lean and obese mice

**Figure 4.**
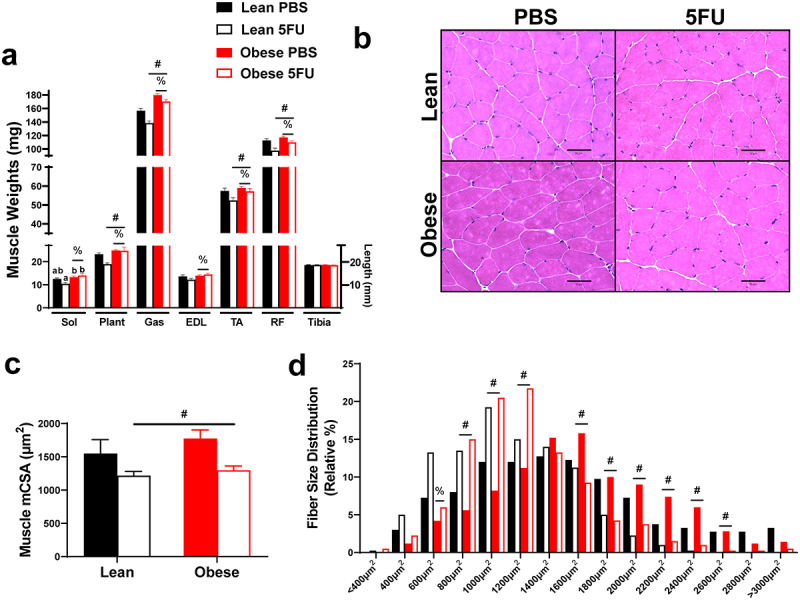
**Skeletal muscle mass and cross-sectional area**. a) Hindlimb (soleus, plantaris, gastrocnemius, tibialis anterior, extensor digitorum longus, rectus femoris) muscle weights taken at tissue excision given in milligrams (mg). Tibias were cleared of excess tissue and lengths measure in millimeters using vernier calipers. b) Representative hematoxylin and eosin (H&E) images of frozen tibialis anterior (TA) 10–12 μm cryosections. c) Mean myofibrillar cross sectional area (CSA) of the TA calculated from H&E stains given in micrometers (μm^2^). d) Distribution of TA cross sectional area calculated from H&E stains given as a relative distribution (%) across increasing μm^2^ sizes. Values are means ± SEM. Two-way ANOVA and LSD post hoc and multiple comparisons. #Indicates main effect of 5FU. %Indicates main effect of Obese. Different letters signify statistically significant differences with an interaction. Significance was set as *p* < .05.

Given the observed loss in body weight and relevance of cachexia for patient quality of life and survival, we sought to examine the impact of obesity and 5FU on skeletal muscle mass. There were main effects of Obese to have increased Sol (p = .002), Plant (p = .002), Gas (p < .0001), EDL (p = .021), TA (p = .035), and RF (p = .004) weights with an additional main effect of 5FU to reduce the weights of the Plant (p = .046), Gas (p < .0001), TA (p = .025), and RF (p = .0003; Figure 4a). A significant interaction (p = .036) was observed and revealed that within 5FU, Obese had increased Sol weight compared to Lean (Figure 4a). Tibia length was similar across all groups. There was a main effect (p = .009) of 5FU to have decreased mean myofibrillar cross-sectional area (CSA) in the TA irrespective of weight status, further demonstrating obesity did not protect against 5FU-induced cachexia (Figure 4b, c). This was also reflected in the fiber size distribution with a leftward shift in fiber size in both Lean and Obese demonstrated by main effects (p < .05) of 5FU to increase percent of fibers at 800 µm^2^, 1000 µm^2^, and 1200µm^2^, while decreasing percent of fibers at 1600µm^2^, 1800µm^2^, 2000µm^2^, 2200 µm^2^, 2400 µm^2^, 2600 µm^2^ (Figure 4d).

### Obesity perturbs skeletal muscle monocyte and macrophage abundance and phenotype

**Figure 5.**
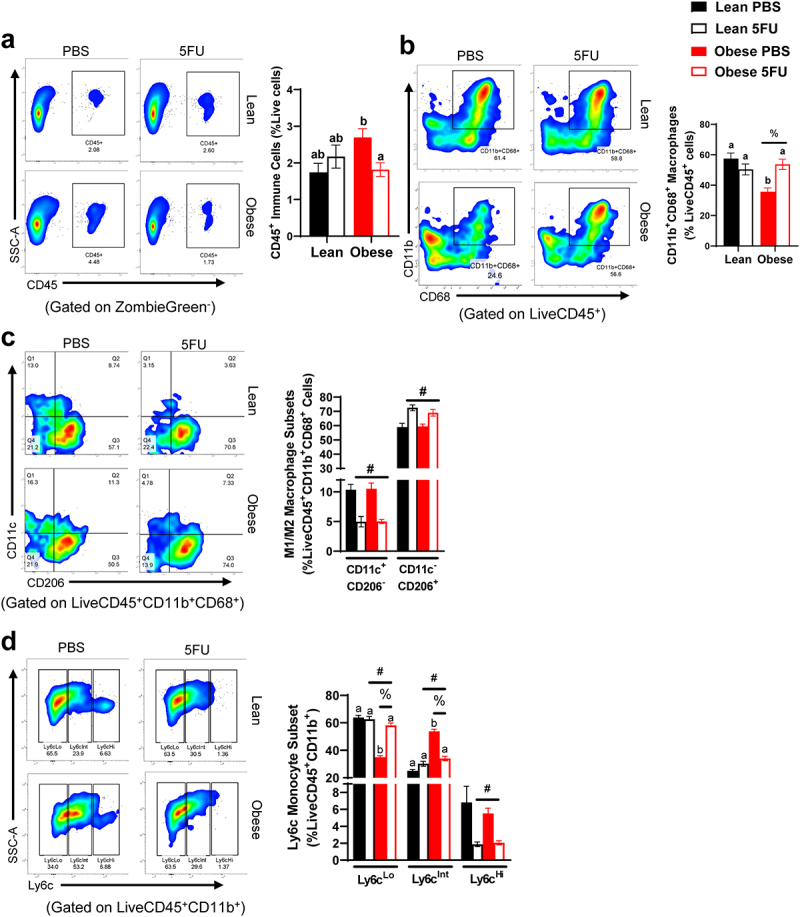
**Skeletal muscle immune cells**. a) Representative flow plots of Cluster of Differentiation (CD) 45^+^ immune cells in all groups and quantified results shown as percent abundance among live cells. b) Representative flow plots of CD11b^+^CD68^+^ macrophages in all groups and quantified results shown as percent abundance among LiveCD45^+^ cells. c) Representative flow plots of CD11c x CD206 in all groups and quantified results shown as the percent abundance among LiveCD45^+^CD11b^+^CD68^+^. Quadrants indicate 4 distinct quantifiable populations, Q1: CD11c^+^CD206^−^, Q2: CD11c^+^CD206^+^, Q3: CD11c^−^CD206^+^, and Q4: CD11c^−^CD206^−^. d) Representative flow plots of Ly6c^Lo/Int/Hi^ monocytes/macrophages in all groups and quantified results shown as the percent abundance among LiveCD45^+^CD11b^+^ cells. Tertiles indicate 3 quantifiable populations, Ly6c^Lo,^ Ly6c^Int,^ and Ly6c^Hi^. Values are means ± SEM. Two-way ANOVA and LSD post hoc and multiple comparisons. #Indicates main effect of 5FU. %Indicates main effect of Obese. Different letters signify statistically significant differences with an interaction. Significance was set as *p* < .05.

We have previously shown that one cycle of 5FU disrupts the immunophenotype of skeletal muscle,^9^ so we sought to examine if obesity impacts skeletal muscle susceptibility to 5FU-induced skeletal muscle leukopenia. Skeletal muscle flow cytometry gating rubrics are shown in Supplemental figures 3 and 4. While we have previously shown that one cycle of 5FU decreases the total abundance of CD45^+^ immune cells in skeletal muscle,^9^ this was not apparent after three cycles in Lean mice. While no main effects were observed, we did observe a significant interaction (p = .019) which revealed that within Obese, 5FU decreased CD45^+^ immune cells compared to PBS (Figure 5a). There was a main effect (p = .012) for Obese to have decreased relative abundance of CD11b^+^CD68^+^ macrophages with an observed significant interaction (p = .0012) revealing that within PBS, Obese had reduced CD11b^+^CD68^+^ macrophages compared to Lean and within Obese, 5FU had increased CD11b^+^CD68^+^ macrophages compared to PBS (Figure 5b). There were main effects of 5FU to have decreased M1-like CD11c^+^CD206^−^ macrophages (p < .0001) and increased M2-like CD11c^−^CD206^+^ macrophages (p < .0001; Figure 5c). Interestingly, there were main effects for both 5FU and Obese to impact Ly6c^Lo^ (p < .0001) and Ly6c^Int^ (p < .0001) monocytes with an observed interaction (p < .0001) (Figure 5d) revealing that within Obese, PBS had reduced Ly6c^Lo^ monocytes compared to 5FU, and similarly within PBS, Obese had reduced Ly6c^Lo^ monocytes compared to Lean (Figure 5d). Conversely, within Obese, PBS had increased Ly6c^Int^ monocytes compared to 5FU and within PBS, Obese had increased Ly6c^Int^ monocytes compared to Lean (Figure 5d). Lastly, there was a main effect (p = .0002) of 5FU to reduce Ly6c^Hi^ monocytes (Figure 5d).

### 5FU decreases inflammatory cytokine gene expression only in obese mice

**Figure 6.**
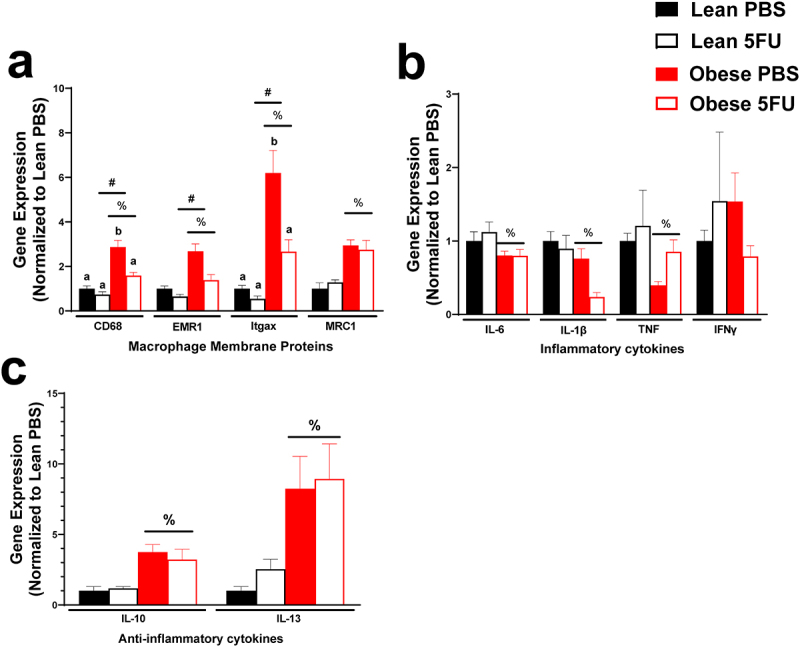
**Skeletal muscle inflammatory gene expression**. a) Relative gene expression of macrophage surface proteins, cluster differentiation (CD) 68, EGF-like module-containing mucin-like hormone receptor-like 1 (EMR1; F4/80), Integrin, alpha X (Itgax; CD11c), and Mannose receptor C-type 1 (MRC1; CD206). b) Relative gene expression of inflammatory cytokines, Interleukin (IL) 6, IL-1β, tumor necrosis factor, and interferon (IFN) γ. c) Relative gene expression of anti-inflammatory cytokines IL-10 and IL-13. Gene values were normalized to vehicle treated controls and compared to five reference targets. Values are means ± SEM. Two-way ANOVA and LSD post hoc and multiple comparisons. #Indicates main effect of 5FU. %Indicates main effect of Obese. Different letters signify statistically significant differences with an interaction. Significance was set as *p* < .05.

Continuing to the inflammatory environment of skeletal muscle, we examined gene expression of critical macrophage surface proteins, CD68, EMR1 (F4/80), Itgax (cd11c), and MRC1 (CD206) as well as pro- (IL-6, IL-1β, TNFα, and IFNγ) and anti- (IL-10 and IL-13) inflammatory cytokines. There were main effects of Obese to have increased CD68 (p < .0001), EMR1 (p = .0005), Itgax (p = .0002), and MRC1 (p < .0001) (Figure 6a). Additionally, there were main effects of 5FU to have decreased CD68 (p = .0055), EMR1 (p = .011), Itgax (p = .026), but not MRC1 (p = .89) (Figure 6a). There was a significant interaction (p = .033) where within PBS, Obese had increased CD68 compared to Lean and then within Obese, 5FU had reduced CD68 compared to PBS. Also, there was a significant interaction (p = .049) where within PBS, Obese had increased Itgax compared to Lean and then within Obese, 5FU had reduced Itgax compared to PBS (Figure 6a). There were main effects of Obese to have reduced expression of pro-inflammatory genes IL-6 (p = .012), IL-1β (p = .003), and TNFα (p = .03) (Figure 6b) and there was a main effect of 5FU to have reduced IL-1β (p = .03) (Figure 6b). Interestingly, there were main effects for Obese to increase anti-inflammatory cytokines IL-10 (p = .0007) and IL-13 (p = .0065) genes, which were not affected by 5FU (p = .77, p = .63) (Figure 6c).

### 5FU decreases adipocyte size and adiposity only in lean mice

**Figure 7.**
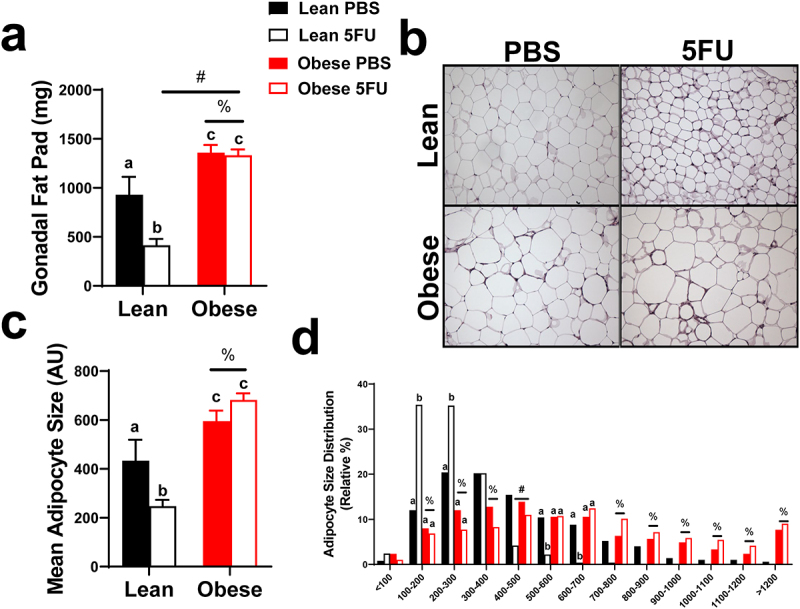
**Adipocyte size**. a) gonadal fat pad weight taken at tissue excision given in milligrams (mg). b) Representative hematoxylin and eosin (H&E) images of fixed gonadal adipose tissue. c) Mean adipocyte size calculated from H&E stains given in arbitrary units (AU). d) Distribution of adipocyte size calculated from H&E stains given as a relative distribution (%) across arbitrary sizes. Values are means ± SEM. Two-way ANOVA and LSD post hoc and multiple comparisons. #Indicates main effect of 5FU. %Indicates main effect of Obese. Different letters signify statistically significant differences with an interaction. Significance was set as *p* < .05.

Lastly, we sought to determine if the wasting effects of 5FU were specific to lean tissue by examining the impact of obesity and 5FU on adipose tissue. As expected, there were main effects of Obese to have greater adipose tissue weight (p < .0001), (Figure 7a) and adipocyte size (p < .0001), (Figure 7b, c, d). Conversely, there was a main effect (p = .01) of 5FU to have decreased gonadal fat pad weight. Significant interactions were discovered for gonadal fat weight (p = .02) and adipocyte size (p = .01); within Lean, 5FU had decreased fat pad weight and adipocyte size compared to PBS and within 5FU, Obese had increased fat pad and adipocyte size compared to Lean (Figure 7a, c).

### 5FU did not impact adipose tissue inflammation however obesity increases inflammatory gene expression

**Figure 8.**
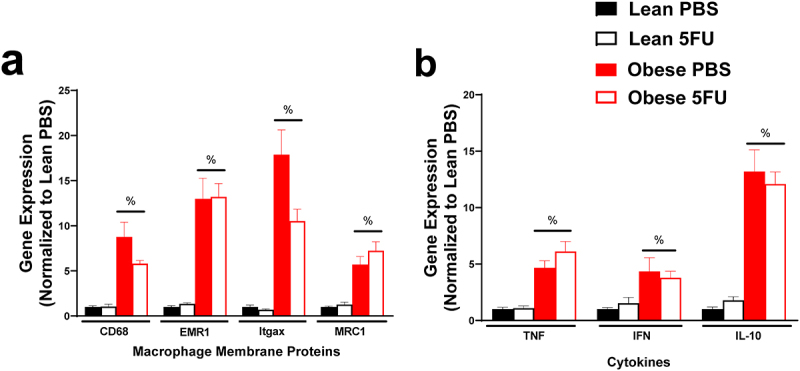
**Adipose tissue inflammatory gene expression**. a) Relative gene expression of macrophage surface proteins, cluster differentiation (CD) 68, EGF-like module-containing mucin-like hormone receptor-like 1 (EMR1; F4/80), Integrin, alpha X (Itgax; CD11c), and Mannose receptor C-type 1 (MRC1; CD206). b) Relative gene expression of inflammatory cytokines, tumor necrosis factor (TNF) α, and interferon (IFN) γ, and anti-inflammatory cytokine IL-10. Gene values were normalized to vehicle treated controls and compared to five reference targets. Values are means ± SEM. Two-way ANOVA. %Indicates main effect of Obese. Significance was set as *p* < .05.

We have previously shown that 5FU alters skeletal muscle and colon tissue inflammation and inflammatory signaling,^9,10^ but little is known regarding 5FU’s impact on adipose tissue. As expected, there were main effects of Obese to have increased macrophage genes CD68 (p < .0001), EMR1 (p < .0001), Itgax (p < .0001), and MRC1 (p < .0001) (Figure 8a) and increased cytokines TNFα (p < .0001), IFNγ (p = .013), and IL-10 (p < .0001) (Figure 8b); however, the inflammatory status of adipose tissue with obesity did not appear to be impacted by 5FU (i.e. there was no main effect of 5FU).

## Discussion

Improving our understanding of the consequences of cancer therapy will foster the development of novel cancer treatments and therapeutics to mitigate anti-cancer treatment toxicities. Importantly, not all cancer patients have the same preexisting conditions/comorbidities, nor do they respond consistently to the same treatments. Therefore, the current study aimed to understand the impact of obesity on the off-target effects of chemotherapy. Our results demonstrated that Obese mice had reduced liver *dpyd* enzyme gene expression and DPD protein – the enzyme responsible for 5FU catabolism – which likely contributed to reduced survival and increased systemic and skeletal muscle immune cell cytotoxicity with 5FU. Further, while our mice were dosed based on relative lean mass, 40% of the Obese mice were unable to sustain 3 cycles of 5FU. Although an obesity paradox has been postulated, the findings from the current study highlight that an obese phenotype was not protective or beneficial and even worsened several 5FU-associated toxicities and survival. Importantly, this suggests reconsidering the current treatment paradigm for 5FU administration and underscores the importance of personalized medicine. While the current study aimed to examine the impact of obesity on chemotherapy toxicities alone, the results from the current study highlight a need for additional clinical examinations and more mechanistic work using tumor-bearing mice.

Currently, the literature on the impact of weight status on chemotherapy-induced toxicities is sparse and equivocal. Evidence demonstrating improved survival with increasing body mass indexes (BMI) has led to the emergence of the Obesity Paradox – obesity is linked with an increased risk for developing cancer but also linked with better survival following a cancer diagnosis–; however, this hypothesis is contentious.^22,26,27^ One potential explanation for this paradox is patients having ‘more weight to spare’ which would offset/delay the severity of cachexia.^24^ Conversely, limiting chemotherapy dosages for patients with larger body surface areas (BSA; 2.0–2.2 m^2^) is common practice clinically, despite a push to provide larger patients with full BSA dosages.^28^ This comes from evidence showing that capping chemotherapy dosing did not reduce treatment toxicities or improve prognosis.^28–30^ This recommendation itself is paradoxical given the proposed improved survival observed with higher BMIs. Furthermore, it is not abundantly clear if the improved survival with a high BMI is consistent when dosages are capped. To the best of our knowledge, the current study design is the first of its kind and demonstrated that obesity was not protective against 5FU toxicities, but rather reduced survival and increased several toxicities. While certain limitations in our study exist (i.e., animal model, severity of obesity, examination of non-tumor bearing mice, and limited mechanistic insight), it is an important first step in the field and points to a need for more clinical and preclinical investigations into the impact of obesity on the efficacy and susceptibility to toxicity of common anti-cancer drugs.

A reduction in blood cell counts is among the most common and detrimental side effects of anti-cancer treatments. Here, we show that while white blood cells are dramatically reduced after one cycle of 5FU, these counts were returned following three cycles in Lean mice. Interestingly, Obese mice had reduced white blood cells following both one and three cycles. Our group and others have previously shown that 5FU induces white blood cell loss and neutropenia by depleting immune cells in bone marrow and inducing cell cycle arrest.^9^ Additionally, this toxicity has been linked to DPD enzyme activity and catabolism of 5FU.^14,31^ We have extended this to show that livers from Obese mice have reduced *dypd* mRNA expression and DPD protein expression concomitant with reduced white blood cells throughout three cycles of 5FU. Interestingly, Lean mice increased DPD protein expression consistent with reports of improved 5FU clearance and reduced 5FU toxicity and efficacy throughout several cycles of chemotherapy. This increase was not observed with Obese which would help explain the sustained cytotoxicity. It is possible that this cytotoxicity may improve 5FU’s anti-cancer efficacy; however, this requires additional investigation involving tumor-bearing mice.

The progression of NAFLD with obesity is well established, yet, whether obesity induced NAFLD contributes to increased chemotherapy-induced toxicities is not as clear. In the current study, we show that livers from Obese mice, regardless of 5FU cycle, have increased fibrotic collagen gene, Col3a1, with reduced *dpyd* expression, reduced DPD protein, and increased liver lipid content. While a mechanistic link cannot be made from the current study, to the best of our knowledge, we are the first to demonstrate reduced DPD enzyme gene and protein expression with obesity. It is currently unknown if obesity is accompanied by reduced liver DPD in the clinic. Our result highlights a need for future studies understanding the impact of weight status, liver function, and overall metabolism on chemotherapy pharmacology and toxicity. Additionally, our results further support the narrative that dosing 5FU should be based on DPD enzyme expression or activity.^14^ Further, there is a concerning dearth of clinical information on the impact of obesity on uracil and/or 5FU metabolism.

While a loss of white blood cells is clinically vital to indicate chemotherapy dose modification and the patient’s susceptibility to further complications with treatment, a loss of red blood cells contributes to anemia and functional deficits that plague cancer patients throughout treatment and often in the years following. In the current study, we show that obesity had no direct impact on 5FU-induced loss of red blood cells; however, there was an effect of 5FU cycle as red blood cells continued to drop after one cycle to be further reduced after three cycles, which has been reported in the clinic.^32^ 5FU-induced anemia likely contributes to the complexities of cancer and chemotherapy-related fatigue.^33–38^ The etiology of 5FU-induced anemia is not well understood but is most often associated with 5FU’s toxicity to the hematopoietic system.^9,12,32^ Further, anemia has been identified as an important influence in the pathology of unintentional wasting with cancer, termed cachexia.^39,40^ Our results demonstrate that obesity was not protective against 5FU-induced anemia.

Cachexia is the unintentional loss of body weight, particularly lean mass, that accompanies chronic disease.^39^ The etiology of cancer-cachexia is complex, and depends on the underlying cancer, the patient’s age and comorbidities, and the treatment strategy. While 5FU containing therapies, FOLFOX/FOLFIRI, have been demonstrated to induce cachexia,^41–43^ 5FU monotherapy’s impact on muscle mass has appeared less severe.^9,11^ Despite this, the results from the current study demonstrate that three cycles of 5FU was sufficient to induce muscle mass loss and myofibrillar CSA loss in both Lean and Obese mice. While these results do not attempt a mechanistic explanation for 5FU-induced atrophy, 5FU has been shown to suppress phospho-Akt (S473), increase phospho-P38, and reduce myoblast cell viability which all have been demonstrated to promote muscle mass loss and myopathy with cachexia.^11,42^ Additionally, 5FU has been demonstrated to disrupt muscle mitochondria which also can contribute to muscle mass loss.^44,45^ It is also likely that the dose and dosing regimen contributes to the equivocal results pertaining to 5FU-induced muscle mass loss as there is currently no established model for chemotherapy dosing in preclinical study designs. Further, while Obese mice had increased muscle mass, this was not protective against 5FU induced muscle mass loss. Unfortunately, while muscle mass was not spared, the maladaptive increase in adipose tissue size and inflammation was not affected by 5FU. Together, this suggests the weight loss observed with obesity is even more catastrophic given the loss of lean mass and sparing of fat mass which would exacerbate function loss and metabolic dysfunction. Future studies are needed to examine the mechanistic overlap between obesity, cancer, and 5FU-induced skeletal muscle perturbations related to mitochondria or overall metabolic health.

Although the impact of 5FU on muscle mass remains equivocal, we confirm our previous findings that 5FU-induced cytopenia extends beyond circulation to impact skeletal muscle’s immune cell pool.^9^ Indeed, we extend our previous findings to show that while one cycle of 5FU reduced total skeletal muscle CD45^+^ immune cells (preplanned t-test, p = .036, data not shown), this returned to baseline following three cycles. Interestingly, Obese mice without 5FU had elevated CD45^+^ immune cells, Ly6c^Int^ monocytes/macrophages, and inflammatory gene expression, indicating increased immune cells and baseline inflammation. This increase in immune cells did not correspond to an increase in CD11b^+^CD68^+^ macrophages; however, these values are representative of relative abundance (%) rather than total counts. Increased macrophages and T-cells have been previously reported in skeletal muscle with obesity, while mast cells, eosinophils, neutrophils, B cells, NK cells were not changed with obesity.^46^ We extend our previous findings to show that 5FU reduced monocytes and macrophages, particularly reducing inflammatory CD11b^+^Ly6c^Hi^ and CD68^+^CD11c^+^CD206^−^ cells after three cycles. Together, our data demonstrate that obesity does not protect against 5FU-induced leukopenia, but rather demonstrates certain evidence of perturbed tolerance based on altered baseline (Obese PBS). One key physiological role of immune cells in skeletal muscle is the repair of muscle following damage, whether pathological or exercise induced. We have previously shown that exercise can improve muscle mass and function during cancer;^36,47^ however, whether these adaptations are maintained with concomitant cytotoxic chemotherapy remains elusive.^48^

As previously stated, the current study has important limitations and delimitations. Given the lack of preclinical investigations into the impact of obesity on chemotherapy toxicities, it was important to examine these two key variables prior to its examination with a third – tumor presence. This comes with an important limitation that the presence of a tumor will elicit its own metabolic and inflammatory perturbations. Recently, the overlap of chemotherapy-toxicities and cancer associated wasting has been eloquently examined;^41,43,49^ however, additional work is needed. Introducing high fat diet into tumor-bearing mice to counteract cachexia has been previously examined;^50^ however, clinical investigations have determined traditional nutritional interventions cannot prevent or improve cachexia.^39^ To the best of our knowledge, there are no preclinical studies examining the impact of obesity on cachexia progression when obesity is achieved prior to tumor or chemotherapy exposure; however, clinical investigations have highlighted that obese cancer patients experience a loss of muscle mass without particular changes to total body weight.^51,52^ The current study is also limited in its mechanistic insight. Additional work is needed to address causal effect of obesity on DPD deficiency as well as the causal link between the drop in DPD with obesity and chemotherapy toxicities. While important gene mutations have been extensively characterized,^12,14^ there is a dearth of information on the behavioral or environmental determinants of liver DPD expression.

## Conclusions

The obesity paradox is likely more nuanced, as has been suggested,^25^ and a gradation is important when delineating the impact of weight status as the animals in the current study were severely obese (~60 g BW, ~50% body fat). Further, the underlying condition and cancer-type appear to play a critical role as well.^53^ The current study demonstrated that obese mice lost similar muscle mass and greater body weight with 5FU when compared to lean mice. Obese mice also had perturbed immune cell populations compared to their lean counterparts. Importantly, obese livers are likely unable to metabolize 5FU efficiently, leading to prolonged and increased toxicities. These data suggest obese mice are not protected against 5FU-induced cachexia or skeletal muscle leukopenia and are more susceptible to certain 5FU-induced cytotoxicity likely due to obesity-induced liver perturbations. Further, these results urge additional work in this realm given the increasing prevalence of obesity and its relevance to cancer.

## Materials and methods

### Animals

Thirty male C57BL/6 mice were purchased from Jackson Laboratories at 8 weeks of age and housed in the Department of Laboratory Animal Resources at the University of South Carolina. Mice were group housed (5/cage) and kept on a 12:12 h cycle. Animals were placed on a purified AIN-76A (Bio-Serv; cat#: F1515; Pro: 18.2%, Fat: 5.1%, Carb: 65.2%, 3.79 kcal/gram) diet and allowed to acclimate to the new facility for 3 weeks. Mice were then given a high fat diet (HFD; Bio-serv, Cat#: F3282; Pro: 20.5%, Fat: 36.0%, Carb: 35.7%, 5.49 kcal/gram) for ~20 weeks (Obese) or maintained on AIN-76A diet (Lean). Mice were then randomized into two groups within each weight status, three cycles of 5FU (n = 5 Lean; n = 10 Obese) or PBS vehicle control (n = 5 Lean; n = 10 Obese). A separate cohort of 20 mice (n = 10 Lean; n = 10 Obese) were subjected to 1 cycle of 5FU to validate consistent results across publications.^9^ Body weights were measured weekly, and mice were monitored for signs of distress (grooming, temperature, anorexia, and tremors). Animals that lost more than 15% body weight were considered ‘end-point’ according to our Institutional Animal Care and Use Committee (IACUC) policy. Survival was examined in all groups (N = 5 Lean PBS, n = 5 Lean 5FU, N = 10 Obese PBS, N = 10 Obese 5FU). Animals were given food and water *ad libitum* throughout the duration of the study. All animals were fasted 5 h prior to tissue collection. Mice were anesthetized with isoflurane and hindlimb skeletal muscles and select organs were carefully dissected, weighed, and either snap frozen in liquid nitrogen or placed in the appropriate buffers for flow cytometry analysis. Animals were euthanized by a cardiectomy following tissue excision while still under anesthesia. All animal experiments were approved by the University of South Carolina’s IACUC.

### 5-FU administration

We have previously demonstrated that male C56BL/6 mice can sustain three cycles of 5FU dosed at 35 mg/kg of body weight.^10^ We then back calculated the dosages of 5FU for lean mass to be ~40 mg/kg of lean mass. Therefore, prior to the initiation of the treatments in the current study, all mice underwent dual-energy X-ray absorptiometry (DEXA; Lunar PIXImus) analysis to determine body composition. Total lean mass percentage was calculated from measured lean mass and total body weight. At the beginning of each dosing cycle, 5FU was solubilized in phosphate buffered saline (PBS; 7.4pH) at 4.0 mg/mL under gentle agitation at 37°C, sterile filtered (0.2 µm) and stored at 4°C for no longer than 7 days. Mice were then given i.p. injections of 5FU at 40 mg/kg of lean mass. Mice were subjected to DEXA analysis once prior to each dosing cycle to adjust for changes to relative lean mass percentage. The dose of 5FU was then calculated daily based on body weight corrected from relative lean mass percentage. Mice in the 3-cycle group were injected with 5FU for 1) 5d, rest 9d, 2) 5d, rest 9d, 3) 5d and euthanized 48 h following the last 5FU injection. Lean and obese vehicle controls were given i.p. injection of PBS (Lean/Obese PBS). Mice in the one cycle group were injected with 5FU for 5d and euthanized 48 h following the last 5FU injection.

### Tissue collection

At the completion of the treatment period, animals were fasted for 5 h, anesthetized with ~2% isoflurane with 2 L/min O_2_. While sedated, the hindlimb muscles, soleus (Sol), plantaris (Plant), gastrocnemius (Gas), tibialis anterior (TA), extensor digitorum longus (EDL), and quadriceps (Quad) were excised, weighed, and either snap frozen in liquid nitrogen or placed in DMEM (Quad) on ice. Additionally, the rectus femoris (RF) was carefully teased away from the vastus lateralis, medialis, and intermedius and weighed prior to being placed in DMEM. Following skeletal muscle excision, the gonadal fat pad, spleen, kidney, liver, and heart were excised, weighed, and place either in 10% neutral buffered formalin or snap frozen in liquid nitrogen.

### Blood analysis

For insulin measurements blood was collected with plain capillary tubes (Kimble; Cat# 2502) prior to anesthesia exposure via the tail vein. Serum insulin concentrations were analyzed according to the manufacturer’s instructions using an insulin ELISA kit (Mercodia, Winston Salem, NC). Blood was then also collected retro-orbitally prior to euthanasia while the mouse was sedated with 2% isoflurane, placed in an EDTA coated vacutainer, and stored briefly on ice until analysis. A complete blood count was performed using the VetScan HMT (Abaxis, Union City, CA, United States) for determination of white blood cells and subsets including lymphocytes, monocytes, and neutrophils, as well as red blood cells, hemoglobin, and hematocrit.

### RNA isolation and RT PCR

RNA isolation, cDNA synthesis, and RT-PCR were performed as previously described.^9^ RNA isolation from liver and rectus femoris was performed using TRIzol (Life Technologies; Cat# 15596018), isopropanol (MPbiomedicals; Cat# 194006), and chloroform (Fisher Chemical; Cat# C298). RNA isolation from adipose tissue was performed using the RNeasy Lipid Tissue Mini Kit (Qiagen; Cat# 74804) according to the manufacturer’s instructions. RNA sample quality and quantities were verified using a Nanodrop One Microvolume UV-Vis Spectrophotometer (Thermo Fisher Scientific, Waltham, MA, United States) and determined to be of good quality based on A260/A280 and 260/230 values (>1.8) prior to cDNA synthesis using High-capacity Reverse Transcriptase kit (Applied Biosystems, Cat# 4368814). Probes from DPYD, TGFβ, Col3a1, Col1a1, Emr1 (F4/80), CD68, Itgax (CD11c), Mrc1 (CD206), TNFα, IFNγ, IL-10, IL-1β, IL-6, and IL-13 as well as housekeeping genes Hmbs, Hprt, B2M, TBP, H2afv, and 18s were purchased from Applied Biosystems. Quantitative RT-PCR analysis was carried out as per the manufacturer’s instructions using Taq-Man Gene Expression Assays on a Qiagen Rotor-Gene Q. Data were normalized to vehicle treated controls and compared to five reference targets (Hmbs, B2M, TBP, H2afv, and 18s), which were evaluated for expression stability using GeNorm.

### Western blotting

Liver protein isolation and western blotting was completed as previously described.^54^ Frozen liver tissue (n = 5–10) was homogenized in Mueller buffer containing a protease inhibitor cocktail (SigmaAldrich, St. Louis, MO). Total protein concentration was determined by the Bradford method. 20 μg of crude liver protein homogenates were separated by SDS-PAGE using precast 8–16% SDS-polyacrylamide gels (Bio-rad Cat#:5671105). Once proteins were fractionated, the proteins were electrophoretically transferred to a PVDF membrane with a Genie Blotter (IDEA Scientific, Minneapolis, MN). Membranes were then stained with Ponceau S solution to verify equal protein loading and transfer efficiency. Membranes were washed and blocked for 1 hr in 5% nonfat milk in Tris-buffered saline-0.1% Tween-20 (TBST). Primary antibody for DPD (Abcam, Cat#: ab180609) was diluted 1:1000 in 5% milk TBST. Membranes were then incubated in primary antibody overnight at 4°C with gentle agitation. Membranes were washed and incubated in secondary anti-rabbit (Cell Signal, Cat# 7074) diluted 1:3000 in 5% milk TBST for 1 hr at room temperature. An enhanced chemiluminescent substrate for detection of horseradish peroxidase was used to visualize the antibody–antigen interaction, which was visualized using the Syngene: G-Box. Images were analyzed by determining the integrated optical density of each band using ImageJ (NIH software).

### Flow cytometry

Isolation of muscle immune cells and flow cytometry were performed as previously described.^9^ A single-cell suspension of cells was prepared in PBS and stained for dead cells using Zombie Green™ (BioLegend, San Diego, CA; Cat#: 423111) following the manufacturer’s instruction. Cells were then suspended in flow buffer (5% FBS, 100 mM Hepes, 2 mM EDTA) and incubated with Fc-block against CD16 and CD32 (BioLegend, Cat#101302) before fluorescent staining. Cells were split into two panels and stained with fluorescently labeled antibodies from BioLegend. The two panels run were 1) “Monocyte Infiltration” CD45 (PE; Cat#:103106), CD11b (APC; Cat#:101211), CD64 (PE/Cy7 Cat#:139314), CD68 (APC/Cy7 Cat#:137024), and Ly6c (PerCP/Cy5.5; Cat#128012) and then 2) “Macrophage Polarization” CD45 (PE/Cy7 Cat#:103114), CD11b (APC; Cat#:101211), CD68 (APC/Cy7 Cat#:137024), CD206 (PE; Cat#141706), and CD11c (PerCP/Cy5.5; Cat#:117328) – ZombieGreen™ (Live/Dead) emits in the FITC detector. Cell markers were selected based on the current understanding of skeletal muscle monocyte/macrophage populations.^9,55,56^ Cells were washed with PBS and then resuspended in flow buffer for analysis. Cell populations were measured using a FACS Aria II cell sorter and analyzed using FlowJo V10.8.1 (BD Biosciences, Ashland, OR, United States). Prior to FlowJo analysis, spectral compensation was performed using Invitrogen UltraComp eBeads™ (Life technologies, Carlsbad CA, United States). Single stained and Fluorescence Minus One (FMO) controls were used to set appropriate gates. A total of 10^6^ isolated cells per mouse were analyzed.

### Histology

Morphology of liver, adipose tissue (gonadal fat pad), and skeletal muscle (TA) was examined histologically. For liver and adipocyte morphology, a portion of the liver and gonadal fat pad, respectively, was excised and placed in 10% neutral buffered formalin (VWR; Cat#: 16004–128) for 18 h, washed with 70% ethanol, and then paraffin embedded. 5–7 μm sections were cut and slides were deparaffinized by xylene, rehydrated with decreasing concentrations of ethanol in water, and then stained with Hematoxylin and Eosin. Images (20x – adipose; 4x/20x – liver) were taken using a Nikon E600 microscope. Adipocyte area was measured using ImageJ (NIH; Bethesda, MD) by measuring the circumference of 150–350 adipocytes (n = 5–10/group). For skeletal muscle morphology, the TA was carefully excised, cut ~1 mm proximal to the mid belly and flash frozen in liquid nitrogen. 10 μm sections were cut using a Zeiss Microm 505HN Crystat (Oberkochen, Germany) at −24°C and slides were kept at −80°C until staining. Slides were fixed in ice cold acetone for 10 minutes and stained with Hematoxylin and Eosin. 20 and 40x images were taken using a Keyence BZX800 microscope. Myofibrillar cross sectional area (CSA) was measured using ImageJ by measuring the circumference of 300–700 myofibers/mouse (n = 5/group).

### Statistics

All data is shown as means ± standard error (SEM). Data was graphed using Prism GraphPad (San Diego, CA) and statistical analysis was run with IBM® SPSS® (Armonk, NY). A two-way ANOVA, general linear model – univariate analysis of variance, was used to determine differences between weight status (Lean, Obese) and chemotherapy (PBS, 5FU). Significant main effect differences between groups (weight status or chemotherapy) were assessed. If a significant interaction was achieved, a post hoc multiple comparison analysis using an LSD adjustment to compare dependent variables within and across weight status and chemotherapy was completed to determine where the interaction occurred. In circumstances where main effects were achieved, the values were collapsed before comparisons. Survival analysis was examined using a Montel-Cox log-rank survival probability test. Significance was set a p ≤ .05.

## Supplementary Material

Supplemental Material

## Data Availability

All data generated or analyzed during this study are included in this published article and its supplementary information files.
